# Putting our money where our mouth is? The degree of women-centred family planning in the era of FP2020

**DOI:** 10.3389/fgwh.2023.1148851

**Published:** 2023-05-30

**Authors:** Alice Witt, Eloisa Montt-Maray, Marieme Fall, Elizabeth Larson, Nour Horanieh

**Affiliations:** ^1^ Department of Global Health and Development, London School of Hygiene and Tropical Medicine, London, United Kingdom; ^2^ Department of Population, Family and Reproductive Health, Johns Hopkins Bloomberg School of Public Health, Baltimore, MD, United States; ^3^ Department of Family and Community Medicine, College of Medicine, King Saud University, Riyadh, Saudi Arabia

**Keywords:** family planning, donor, women-centred, measurements, ethics, autonomy, empowerment, discourse

## Abstract

The 1994 International Conference on Population and Development was a landmark moment for the international family planning community, who committed to adopt a women-centred approach to programming—one that would prioritise the reproductive and contraceptive intentions, or autonomy, of individuals over population-level demographic concerns. The FP2020 partnership, established in 2012 and lasting until 2020, also described itself using women-centred language. However, throughout the period of FP2020, critics questioned the extent to which women-centred principles truly defined why family planning programmes were funded and how they were implemented. In this study, we use thematic discourse analysis to examine six major international donors' rationale(s) for funding family planning and the measurements they used to articulate successful programming. We present an overview of the rationales and measurements used by all six donors before offering four case studies to demonstrate divergences in their approaches. Our analysis demonstrates that, although donors described the importance of family planning for fostering women's autonomy and empowerment, they also justified family planning on the basis of demographic concerns. In addition, we identified a misalignment between how donors described family planning programmes—using the language of voluntarism and choice—and how they measured their success—through increased uptake and use of contraceptive methods. We call on the international family planning community to reflect on their true motives for funding and implementing family planning and engage in radically rethinking how they capture programme success, in order to better align their rhetoric with their practice.

## 1. Introduction

Family planning has long been recognised as a valuable instrument in the international development toolkit, with formal programmes offering contraceptive information and methods implemented across the world since the 1950s. Initially, such programmes were a tool of the demographer, who saw family planning as a means of reducing fertility rates to support socioeconomic growth, particularly in the so-called Global South (1–3). However, accepted justifications for funding and implementing family planning programmes have changed substantially over time.

In the late twentieth century, calls grew for the family planning community to adopt a women-centred approach, defined as one that would “*[look] at experiences, values, issues, and information from the point of view of the women whose lives are affected*” (4). Activists argued for a “*fundamental revision*” to existing programmes that would recognise that “*women*”*s empowerment is legitimate and critically important in its own right*” (5). Cairo's 1994 International Conference on Population and Development marked a watershed moment for this movement, culminating in the international community coming to the consensus that:

“*The aim of family-planning programmes must be to enable couples and individuals to decide freely and responsibly the number and spacing of their children and to have the information and means to do so and to ensure informed choices and make available a full range of safe and effective methods*” (6).

The family planning community thus committed to an approach to programming whose core aim is to fulfil the reproductive and contraceptive intentions, and therefore autonomy, of clients and/or to promote women's empowerment.

In 2012, the London Summit on Family Planning marked the launch of the Family Planning 2020 (FP2020) partnership, a global community of family planning actors, including international donors, with the goal of providing modern contraceptives to 120 million additional women and girls by 2020 (7). Notably, FP2020 framed its aim “*to empower women and girls by investing in rights-based family planning*” (7) in the language of the women-centred approach established in 1994.

However, over the period of FP2020, critics questioned the extent to which the promotion of individuals' reproductive and contraceptive autonomy truly defined why family planning programmes were funded and how they were implemented. Some identified that the emphasis on increasing the number of contraceptive users incentivised overpromotion of contraceptive methods, particularly long-acting reversible contraceptives, thereby compromising individuals' reproductive and contraceptive autonomy (8–11). Others argued that because many of the outcome indicators used to measure family planning programme success were designed to monitor population-level demographic changes, they did not and could not capture if and how programmes fulfil individual intentions (3, 12). This is particularly true of the measurement of women with unmet need, defined as those who are sexually active but are not using any method of contraception, and who do not wish to have another child in the next two years (13). This measurement, which captures an individual's reproductive intention but not their intention to use contraception, is widely used to identify target populations for programmes aimed at increasing contraceptive access and use (3, 14–16). As a result, critics have suggested that the shift towards women-centred principles may have operated more at the level of discourse than that of practice (12).

The roll-out of Family Planning 2030 (FP2030) began in 2021, establishing an updated goal for the family planning community:

“*Voluntary modern contraceptive use by everyone who wants it, achieved through individuals’ informed choice and agency, responsive and sustainable systems providing a range of contraceptives, and a supportive policy environment*” (17).

As the family planning community sets out to collectively meet this goal, we want to understand and shed light on how major international actors described the value of family planning over the last decade, and what they understood successful programming to be. In doing so, we aim to foster critical reflection among all actors in the family planning community about how they might better translate women-centred principles into programmatic realities going forwards.

International donors, based largely in the Global North, hold much of the power to design the family planning programme blueprints that are adopted across the sector. For this reason, we chose to examine six major international donors funding family planning programmes in Francophone West Africa (FWA) during FP2020. We used thematic discourse analysis to explore how these donors described their rationale(s) for funding these programmes, which measurements they used to capture programme success, and the degree to which these rationales and measurements aligned with a women-centred approach to family planning. This paper will first present the shared rationales and outcome indicators commonly used by international donors before presenting four in-depth case studies to illustrate diverging approaches.

## 2. Methods

### 2.1. Donor selection

We elected to study six family planning donors who played a major role in funding family planning in FWA during FP2020. We chose to study donors working in FWA due to the high interest and investment in this region during FP2020, mobilised in large part via the Ouagadougou Partnership (OP), a consortium of 9 countries working to reach 2.2 million new contraceptive users by 2020 (18). We took the OP as a proxy for the family planning community in the region and identified active donors by examining publicly available webpages and documents published by the OP on its website. We determined the major donors based on how regularly the OP mentioned their involvement in the partnership and the degree to which the OP presented them as core contributors.

We identified 10 major donors, of which two were private donors, five were bilateral and three were multilateral. We chose to select two donors from each category to examine the perspectives of different donor types (see Table 1). One multilateral donor's website contained a very limited quantity of information about the family planning work they fund. Therefore, to be able to conduct a meaningful analysis for the purposes of this study, we elected to study the remaining two multilaterals identified in the original set of ten. Of the five bilateral donors identified, we selected the two whose involvement the OP cited most frequently on its website and whose contribution it presented as most central to the partnership's work. We studied the two private donors identified in the original set of ten.

**Table 1:** Donor type of the six selected donors.

Donor number	Donor type
1	Multilateral
2	Private foundation
3	Bilateral
4	Multilateral
5	Private foundation
6	Bilateral

All six donors were based in countries in the Global North and had made commitments to FP2020. Our intention is not to critique these donors as individual actors, but to present findings which are relevant for the family planning community as a whole. For this reason, we have chosen not to name the individual donors studied in this paper.

### 2.2. Data sources and extraction

We aimed to explore how these six donors described their rationale(s), or justification(s), for funding family planning programmes. We paid particular attention to the extent to which donors adopted women-centred rationales—ones whose core aim is to promote individuals’ reproductive and contraceptive intentions, and therefore autonomy, and/or women's empowerment—or women-focused rationales, which adhere to these same principles but do not position them at the core of their approach. We considered any justifications for family planning programmes which emphasised the benefits of national fertility rate reduction at the population level, including economic advantages, to be demographic-focused rationales. We also examined the measurements donors used to assess family planning programme success, with a focus on the extent to which outcome indicators captured fulfilment of individuals' reproductive and contraceptive intentions.

We examined written and audio-visual materials publicly accessible on the websites of these donors, including webpages, reports, news articles and videos. We included all sources containing information on the topic of family planning or relevant guiding principles of each organisation. We included sources in both English and French and when the same source was translated in both English and French, we selected the English version for analysis. Sources on the topic of family planning were identified using an exhaustive search on each donor's website using the keywords “Family planning”, “Contraception” and “Planification familiale”. We also manually navigated each donor's website to identify relevant sources. As we found limited publicly accessible material containing details of individual programmes on donors' websites, we used the statistics and cited outcome indicators that donors presented in our existing source materials to identify their preferred measurements of success.

We selected sources published online between January 2013 and April 2021 (inclusive) and available online in April 2021. This timeframe captures the period of the FP2020 partnership from six months after the London Summit on Family Planning until six months before roll-out of FP2030 was due to begin. A total of 132 sources were identified for analysis (see Table 2). We downloaded all sources published on donor websites in PDF format and uploaded them to NVivo Server in their entirety. For all other sources, we extracted all sentences that contained information on or related to family planning or relevant organisational principles into one Word document per donor, and uploaded all six to NVivo Server.

**Table 2:** Number and type of sources per donor.

	Total number of PDF documents	Total number of webpages (excluding videos)	Total number of videos	Total number of sources
Donor 1	4	11	1	**16**
Donor 2	0	13	2	**15**
Donor 3	11	17	3	**31**
Donor 4	15	9	9	**33**
Donor 5	5	13	1	**19**
Donor 6	7	11	0	**18**
Total	**42**	**74**	**16**	**132**

### 2.3. Data analysis

We performed this study using thematic discourse analysis. By Foucault's definition, discourse is the process by which individuals or institutions generate knowledge—or “truth”—about the world, based on their unique perceptions, experiences and agendas, and reproduce this knowledge in regularities of speech and text (19–22). Foucault underlines that institutional processes of meaning-making and meaning-exportation are not value-neutral but instead a means of exerting power in a social world (19, 20). Thematic discourse analysis enabled us to systematically organise information to perceive patterns in how institutions generate meaning around a single phenomenon (21, 23).

We coded the source material using NVivo Server software to organise the data into distinct themes, where each code represented one rationale used to justify funding family planning programmes or captured measurements used to articulate family planning programme success. Two researchers familiarised themselves with all source materials during the process of data extraction and recorded their initial reflections of the rationales and measurements that donors cited. From these reflections, they produced an initial coding framework deductively, which they then applied to the source material until reaching saturation to check for suitability. During this process, they generated further codes inductively and used annotations to note further reflections on the content of the source material and approach of each donor in NVivo. They then updated the coding framework (see Table 3) and applied it to the entirety of the source material using NVivo Server.

**Table 3:** Final coding framework applied to all source material.

Code	Child code
Rationales *The rationale a donor cites for implementing family planning programmes*	Cost-effectiveness *Donor describes family planning programme as a cost-effective intervention*
	Demography *Donor describes demographic concerns in relation to family planning, including mention of fertility rates or population growth or reduction*
	Development *Donor describes family planning as contributing to development and/or international goals*
	Economic growth *Donor describes population-level economic benefits of family planning*
	Environment *Donor describes how family planning can help prevent environmental damage or climate change*
	Feminism *Donor describes implementing family planning out of feminist principles*
	Mortality rates/Health *Donor describes how family planning improves health outcomes or reduces maternal and/or infant mortality rates*
	Multiple justifications *Donor gives several different justifications for family planning within one sentence or list*
	Women's opportunities *Donor describes how family planning improves women's access to educational or economic opportunities*
	Women-centred *Donor describes how family planning is of benefit to women in ways that are outside of educational or economic opportunities*
Measurements *Statistics or programmatic indicators used to articulate programme success*	Unmet need *Statistics or mention of the unmet need indicator*

During analysis, we examined which rationales each donor employed and how each donor interpreted each rationale. We sought to find meaning from the source materials as a collective body of work rather than examining individual words in isolation. We noted each donor's choice of language, the associations made between words and concepts, any contradictions between the various rationales used by each donor, and any rationales or key concepts from the family planning literature which each donor omitted. In addition, we noted which measurements of success each donor cited, any variations in their interpretations of the meaning of each, and the frequency at which each was mentioned, in order to compare between donors.

## 3. Results

Our analysis identified that all six donors justified funding family planning programmes on the basis that they would boost development, economic growth, health, and women's empowerment. However, we found that each donor prioritised different rationales and articulated their conceptualisation of each in different ways. Likewise, though some outcome indicators were common to all donors, emphases put on certain success measurements reflected each donor's unique priorities. To highlight this variation, we will first present the rationales and success measurements that were shared by all six donors, before offering four case studies to illustrate divergences in donor approaches and priorities.

### 3.1. Shared rationales

All donors studied offered multiple rationales for funding family planning, and all six emphasised that family planning programmes are able to address multiple development problems because they have the potential to reduce fertility rates. As one donor described, “*controlling population growth has become a tool for development*” (Donor 6).

All six donors also depicted family planning as a tool to boost economic growth. All six often linked these potential economic advantages to the demographic dividend, where creating a sharp reduction in national fertility rates is thought to boost a country's gross domestic product through increasing the number of working-age people and reducing the number of dependents. Similarly, all but one donor (Donor 5) emphasised that family planning programmes are cost-effective development interventions because they reduce the number of unintended pregnancies, which generates savings in other healthcare sectors and public services. For example, one donor wrote that:

“*Reductions in unintended pregnancies and unplanned births would make improvements in maternal and newborn care more affordable. Providing medical care…for women with unintended pregnancies…as well as for their newborns, currently costs $4.3 billion… In other words, investments in family planning help offset the cost of improving maternal and newborn health care for all women*.” (Donor 4).

The same five donors argued that family planning programmes improve health outcomes and reduce maternal and infant mortality, with four (Donors 1, 2, 3 and 4) explicitly framing family planning as a life-saving intervention.

Each donor also described that family planning programmes support women's empowerment. In particular, they all emphasised that family planning enables women to choose when they fall pregnant, which can increase their access to education and employment opportunities. As one donor stated, with family planning, “*they can stay in high school and then go on to college, they can live their dreams! They have the chance of getting a productive job in society, creating a new company if they want*” (Donor 2).

However, this empowerment rationale was often legitimised on the basis of the benefits it will generate for others “*far beyond women themselves*” (Donor 3). For example, one donor argued that “*Family planning is one of the most cost-effective interventions to improve the human condition, with enduring health and welfare benefits for women, families, and nations*” (Donor 1). Another similarly stated that women's use of family planning will lead to “*increases in household savings and assets, increases in children's schooling, increases in GDP [gross domestic product] growth and reductions in poverty*” (Donor 4).

### 3.2. Shared measurements of success

The outcome indicators that the studied donors most commonly used to capture family programme success were those which measured contraceptive prevalence or use. Examples of such indicators include (modern) contraceptive prevalence rate and couple years of protection. We also found that all six donors used total fertility rate to justify the importance of family planning programmes and used measurements of unmet need to identify target populations. As Donor 5 wrote:

“*The reasons for focusing on Francophone West Africa [for family planning programmes] are straightforward and compelling: it has the highest rate of unmet need for family planning services, the highest total fertility rate, the highest desired fertility, and the highest population growth rate in the world*”.

All six donors used measurements of mortality reductions to demonstrate the success of family planning programmes. Most donors cited reductions in both maternal and infant mortality, though one (Donor 5) only mentioned maternal mortality. In addition, it was common for donors to cite the number of abortions and unintended and/or unwanted pregnancies their programmes had averted, as in the statement:

“*By the end of July 2016, collective efforts helped prevent 1.16 million unintended pregnancies, 368,000 unsafe abortions and 3,890 maternal deaths through giving women education or access to modern contraception*” (Donor 4).

### 3.3. Unique priorities

The following case studies illustrate differences between how each donor conceptualised the primary problem for family planning programmes to solve and the benefits that they can have. They also demonstrate unique points of interest in each donor's use of indicators and how these relate to each donor's rationale(s) for family planning. These case studies are not a summary of each donor's approach overall but are rather aimed at pinpointing some of the key distinctions between them. They cover two multilateral donors, one bilateral donor and one private foundation.

#### 3.3.1. Case study—Donor 1

##### 3.3.1.1. What is the problem for family planning to solve?

Donor 1 articulated the problem for family planning to solve as high fertility rates, describing women with many children as having “*excess fertility*”. This donor presented women's desires to have large families as a “*tough challenge*” and “*entrenched and complex demand-side barriers in the transition to smaller families, poverty reduction and economic growth*”.

##### 3.3.1.2. What are the benefits of family planning?

Donor 1 depicted the principal benefit of family planning as the economic rewards brought by fertility rate reduction. They stated that these rewards would be generated both directly, through realisation of the demographic dividend, and indirectly, through “*human capital accumulation*”. In this latter process, Donor 1 described that family planning improves individuals' educational and health outcomes, thereby making them more economically prosperous citizens.

This donor frequently linked health to “*prosperity*”, as seen in statements such as “*Niger's growth and prosperity depends on a healthy population—children who are ready to learn and men and women who can participate in a productive economy*”. They likewise presented the empowering effect of family planning as valuable for the economic benefits it brings in creating more productive citizens. This was clear in statements such as “*A woman who can choose when, and how many children to have, can stay in school longer and seize economic opportunities for her family—and her country*”.

##### 3.3.1.3. Notable use of measurements?

In line with their emphasis on demographic-focused rationales, Donor 1 frequently cited countries' total fertility rates to highlight the need for family planning programmes. This was particularly common in reference to countries in FWA, as in the comment “*[we] recently approved a complementary population and health project for Niger (the country with the highest total fertility rate in the world, 7.6 children per woman)*”.

#### 3.3.2. Case study—Donor 2

##### 3.3.2.1. What is the problem for family planning to solve?

For Donor 2, international family planning programming was needed to solve technical gaps in “*access*” to contraceptive information and services for women and girls, since “*If we deny access, we are dooming them to a lifetime of poverty*”. In this way, Donor 2 presented issues of access as the primary reason women do not use family planning, such that improved access to services would automatically lead to increased uptake of contraceptives, and subsequently to a range of other benefits. This logic is exemplified in statements such as “*Increased access to contraceptives…results in fewer women and girls dying in pregnancy and childbirth, fewer unintended pregnancies, fewer abortions, and fewer infant deaths*”.

##### 3.3.2.2. What are the benefits of family planning?

Donor 2 emphasised how family planning can reduce “*poverty*”, as is reflected in comments such as “*contraceptives are also one of the greatest antipoverty innovations in history*”*.* In particular, this donor articulated that family planning helps people to help themselves, meaning individuals can “*lift themselves out of poverty and build a better future for their children*”. Similarly, Donor 2 emphasised how family planning supports a woman or girl achieve her “*dreams*” or “*determine her own future*”.

In addition, this donor highlighted that, by reducing the number of births, family planning enables parents to invest greater care and resources in their existing children: they frequently quoted parents making comments such as “*It wouldn’t be fair for me to have another child. I can’t afford to feed the ones I have*”. They also emphasised the positive impact that people using family planning can have on national development:

“*Since the number of young children is relatively smaller, the government and parents are able to invest more in each child's education and health care, which can lead to more economic growth over the long term*”.

##### 3.3.2.3. Notable use of measurements?

Donor 2 regularly used measurements of unmet need to demonstrate the importance of and mobilise support for family planning programmes. This donor was notable in elaborating a narrative around unmet need statistics to give them a human face, in comments such as “*More than 200 million women say they don’t want to be pregnant but aren’t using contraceptives*”. Such narratives were often evocative in tone, as in:

“*This demographic transition can happen in a reasonable period of time only if all women have access to contraceptives. Right now, more than 200 million don’t. For the sake of those women, their children, and their communities, we must meet their needs—and we must do it now.*”.

#### 3.3.3. Case study—Donor 3

##### 3.3.3.1. What is the problem for family planning to solve?

Donor 3 placed great emphasis on how family planning is a “*best buy*” that can cost-effectively address multiple development problems. In 2016, one of the donor's then directors stated that what makes family planning “*so powerful is that you can talk to anybody about whatever their key interest is and usually find a path where family planning can get them to what they are most interested in doing*”.

This donor articulated that by reducing fertility rates, family planning can solve a multitude of problems including climate change, emergence of infectious diseases, water scarcity and food insecurity, declining marine resources, food and chemical waste, poor access to sanitation and risks of civil conflict.

##### 3.3.3.2. What are the benefits of family planning?

Donor 3 closely associated family planning to the realisation of international development goals, particularly the Sustainable Development Goals (SDGs)—the 17 global goals adopted by all United Nations member states in 2015 (24). In fact, meeting the SDGs was portrayed as justification in itself for implementing family planning, as seen in statements such as “*Family planning can accelerate progress across the 5 SDG themes of People, Planet, Prosperity, Peace, and Partnership and is critical to achieving the goals*”. Likewise, this donor saw increased numbers of contraceptive users as a means through which countries can accelerate their trajectory towards development, arguing that “*with amplified and sustained global effort, all countries can achieve levels of demand for family planning met with modern contraceptive methods now enjoyed in OECD ones*”.

This donor also highlighted several ways that family planning supports health outcomes. Firstly, they regularly emphasised that family planning helps to minimise HIV transmission, both due to condoms being a barrier method and because contraception “*prevents unintended pregnancy in women with HIV*”. Secondly, they framed family planning as a tool that fosters “*healthy timing and spacing of pregnancy*”, presenting family planning successes using measurements of unwanted births, mistimed births, high risk births or teenage pregnancies averted. Yet notably, they also included warnings about the number of pregnancies a woman has—“*the risk of maternal death increases as the number of children per woman rises from two to six or more*”—within this discussion of healthy timing.

##### 3.3.3.3. Notable use of measurements?

Donor 3's support of reproductive and contraceptive autonomy was reflected in frequent descriptions that their programmes offer “*voluntary family planning*”. This donor was unique among those studied as it articulated what it meant by voluntarism and informed choice in relation to family planning: in short, being offered access to information and a range of methods and services in order to “*choose voluntarily whether to use family planning or a specific family planning method*”. This donor did not offer any information detailing if and how voluntarism is captured through programmatic outcome indicators, however they did regularly, and distinctively, report programme success through percent demand for family planning met with modern contraceptive methods. By this metric, demand for family planning is calculated as the proportion of the population currently using a contraceptive method added to the proportion with an unmet need (25). In Donor 3's words, this metric

“*reﬂects voluntarism and informed choice—it neither sets contraceptive prevalence nor fertility targets, but rather emphasises the imperative to satisfy individuals’ and couples’ own choices with regard to number and timing of children*”.

#### 3.3.4. Case study—Donor 4

##### 3.3.4.1. What is the problem for family planning to solve?

Donor 4's approach to family planning explicitly sought to address individual- and national-level problems simultaneously. They argued that high fertility rates are a problem in so far as they are a reflection of “*the extent to which people have the power and the means to make their own choices about the number, timing and spacing of pregnancies*”. Donor 4 equated the inability to make these choices to an inability to exercise sexual and reproductive rights. By this logic, family planning supports the realisation of human rights, which they described as essential for national development in and of themselves. This approach is exemplified in statements such as “*Countries where fertility rates have been low for many years are generally more developed. Basic reproductive and other rights are mostly met*”.

##### 3.3.4.2. What are the benefits of family planning?

This donor highlighted how family planning can help people to attain their desired family size and placed great emphasis on family planning's ability to reduce unintended pregnancies, also described as “*unwanted fertility*”. They often cited parents praising family planning for how it helps them to care for the children they already have, such as the father quoted saying “*Even if I had more money, I wouldn’t consider having more kids. Because money wouldn’t give me more time to take care of my kids*.”

However, Donor 4 also drew attention to the challenges faced by countries with low fertility rates, particularly that “*they are ageing rapidly, facing a future where fewer people are in the workforce, and costs related to pensions and health care may be high*”. Likewise, they drew attention to how these challenges can compromise sexual and reproductive rights, such as when “*Gaps in affordable quality childcare…can make it difficult to balance work and family life, leading to people having fewer children than they want*”.

Notably, this donor explicitly stated that women's interests are the priority focus of their family planning work, beyond the number of children they choose to have or the benefits they can have for others. This is exemplified in the statement that:

“*In the end, our success will not just come in reaching what we imagine is ideal fertility. The real measure of progress is people themselves: especially the well-being of women and girls, their enjoyment of their rights and full equality, and the life choices that they are free to make*”.

##### 3.3.4.3. Notable use of measurements?

In line with their rights- and wellbeing-based rationale, Donor 4 also conceptualised family planning as a means of fostering holistic bodily autonomy, which it defined as “*not simply about sexual choices and reproduction. It is about a person's whole self, their dreams and potential in life*”. This donor was unique in engaging in questions of how autonomy could be measured, citing the SDG indicators 5.6.1 and 5.6.2 and how they aim, respectively, to measure autonomous decision-making and the existence of laws and policies that guarantee full and equal access to reproductive healthcare. They also explicitly acknowledged the limitations of current metrics of autonomy, stating that “*these measurements are critical, but they offer only a glimpse into the bodily self-determination of people around the world*”. Distinctively, they called for further work to develop and incorporate metrics which meaningfully capture individuals' bodily autonomy.

## 4. Discussion

Our analysis demonstrates that donors held multiple rationales for funding family planning, including both demographic- and women-focused arguments, during the era of FP2020. We also found that all donors used both demographic- and women-focused rationales simultaneously.

Donors' women-focused rationales emphasised the empowering effects of women being able to choose if and when they fall pregnant. However, although women-focused, donors' rationales were not women-centred: we did not find that fulfilment of individuals' reproductive and contraceptive intentions was the core around which their approaches to family planning were built. In fact, we found that the effect of family planning programmes on women's childbearing was often presented as advantageous in terms of the benefits it would generate for others. In particular, donors emphasised how families being able to choose to have fewer children would free up economic resources and public services, thereby facilitating population-level benefits such as the demographic dividend, increases in gross domestic product and national development. This instrumentalising effect is a characteristic of what Cornwall terms “*empowerment lite*”, the neoliberal empowerment discourse where “*the attention is insistently on what women and girls are able to do for* others*, their agency is implicitly relational, wedded to their families and to the vital role scripted for them as altruistic mothers*” (26). Our findings therefore suggest that the 1994 activists' striving for a family planning which sees that “*women's empowerment is legitimate and critically important in its own right*” (5) may not yet have come to fruition. Further still, we note the strikingly neoliberal character of this approach to realising national development through family planning, which places considerable burden for achieving national prosperity in the hands of individuals, particularly women, and their decisions around child-bearing.

Additionally, the high frequency with which demographic-focused rationales were invoked is notable, given the family planning community's explicit 1994 rejection of population-level priorities guiding programme design (6). In fact, we note that donors’ mobilisation of measurements of high national total fertility rates to articulate the need for family planning programmes appears little changed from the pre-1994 approach. This finding therefore supports the assertions of several critics that, despite being considered by some as the priority of the now distant past, demographic and population-level concerns continue to have considerable influence on why and how family planning programmes are funded and implemented up to the present (9, 12, 27–29). On this basis, it is clear that the conversation on why and how to enact women-centred programming must be (re-)established as a priority of the present, rather than being viewed as a *fait accompli* of the past.

As scholars have shown, prioritisation of demographic concerns directly impacts how family planning success is articulated, specifically that contraceptive use is framed as the primary programmatic objective (2, 3, 12). This reflection is echoed in our finding that contraceptive uptake and prevalence were the primary means through which donors measured family planning programme success during FP2020. However, a growing body of literature highlights how, in practice, this prioritisation can cause an individual's use of a contraceptive method to be considered more valuable than their having chosen to use that method (8–11). With success equated to contraceptive use, programmatic frameworks of this kind consider a woman choosing to decline a contraceptive method an outcome of failure (12). Therefore, family planning programmes that are demographic-focused cannot be truly women-centred since they do not necessarily operate within a value system centred on fulfilling the reproductive and contraceptive autonomy of all individuals.

We did find that donors regularly verbalised the importance of voluntarism, informed choice and autonomy within the contraceptive decision-making process. However, no donors described funding programmes which used outcome indicators to capture whether an individual's use of a contraceptive method was made by a voluntary and informed decision-making process, or if the programme fulfilled the individual's reproductive and contraceptive intentions. We noted that one donor was beginning a nascent conversation around the need to develop and use such indicators, however we found that this was not yet a mainstream conversation in and project of the donor community during the FP2020 period.

This disjuncture suggests that the movement towards women-centred family planning during FP2020 may have taken place more at the level of discourse than that of programming. The inclusion and increasing popularity of women-focused discourses play a critical role in amplifying the voices of individual women within family planning, and there is no doubt considerable strides have been made since 1994. However, real change cannot be effected in the absence of a “*fundamental revision*” (5) to how donors structure programmes and measure their success.

Furthermore, we found that some donors stated or implied that their programmes had offered voluntary family planning by citing measurements of unmet need or percent demand met. However, both of these indicators are built on the assumption that any sexually active woman who does not wish to fall pregnant over the next two years—and therefore has an unmet need—wishes to use a contraceptive method (14, 15). As Speizer et al. show, “*demand satisfied, like unmet need, does not take into consideration whether women are freely choosing to use a method nor if they are using the method of their choice*” (16). As such, in the absence of any measurement of fulfilment of the individual's contraceptive intention, the suggestion that such programmes are grounded in voluntarism cannot be guaranteed. However, donors' rhetorical conflation of these two distinct intentions, and their use of outcome indicators which appear to be women-centred, demonstrates the power of women-focused language in the period of FP2020. Applying the Foucauldian understanding of discourse to our findings shifts them from being simple rationales to being strategic methods of meaning-making and -concealment deployed by those in power to define reality on their terms (19, 20). By this logic, it could be argued that donors instrumentalised women-focused rationales during FP2020 to create “*a ‘normative resonance’ that makes everyone feel good. It aims to please as many people as possible without revealing which meaning they personally favour*” (30). This would follow the noted phenomenon of international development actors using the latest buzzwords to build inward cohesion and outward legitimacy, without necessarily migrating that discourse into practice (31, 32).

The Foucauldian lens is also important as it brings to light the agents and relations of power involved in defining why and how women should be offered family planning. Although it was not our objective to explore what donors understood women-centred family planning to mean during FP2020, it emerged during the course of our analysis that the donors studied—each of which are based in the Global North—had a consistent stance on this: that by giving women control to decide if and when they fall pregnant, family planning improves women's access to educational and employment opportunities, thereby fostering empowerment. On this basis, family planning programmes built on increasing contraceptive use would be inherently empowering. However, this view of the empowering effect of family planning has a distinctly Euro-American character, based on two beliefs about what women want: to have fewer children, and to achieve smaller families through use of a contraceptive method. While there is no doubt that being able to limit their fertility is fundamental to many women's empowerment, anthropological literature is clear that the Euro-American model of empowerment is not applicable to all women (33–35). For example, in many settings, it is through having large families and/or giving birth soon after marriage that women gain agency and status within their communities (36–38). Likewise, many studies demonstrate women's aversion to taking a contraceptive method to control their fertility (39–43). In this way, family planning programmes built around increasing contraceptive use cannot necessarily be women-centred, as they may not be meeting the reproductive and contraceptive intentions of all women. This finding sheds some light on the way in which the concentration of power in institutions in the Global North continues to shape definitions of what women want, to the extent that programmes may not be built in a way that truly meets their needs. We suggest that further exploration of how else women-centred family planning might be conceptualised would be a valuable and fruitful avenue for further research.

## 5. Strengths and limitations

To our knowledge, this study is the first of its kind to use thematic discourse analysis to examine how members of the international community understand the value of family planning and articulate programme success. Our analysis of six donors enables us to demonstrate how individual actors took divergent approaches despite committing to a shared FP2020 agenda. In addition, our identification of many similarities in the approaches of three different types of organisations suggests that our findings have relevance for all actors in the family planning community, not only donors.

This study also has some limitations. Firstly, we only examined data which was publicly available online. Due to a lack of published data on the provenance and sum of funding contributions to family planning programmes in FWA, we were required to determine major donors based only on perceptions of influence and visibility. Likewise, our analysis of donors' preferred outcome indicators was limited by the scarcity of details about the individual programmes that are funded. Secondly, our analysis gives an overview of rationales used in the period of the FP2020 partnership overall but does not examine how each donor's rationales may have changed within that period. Lastly, although we analysed materials in both French and English, we prioritised English versions over French ones (when available) during our analysis process and only included English examples in this manuscript.

## 6. Conclusion

In this study, we used thematic discourse analysis to explore how donors described their rationales for funding family planning in the period of FP2020. We found that donors cited both demographic- and women-focused rationales simultaneously, but did not use women-centred rationales, as they did not place women's reproductive and contraceptive autonomy and/or empowerment at the core of their approach. In addition, we found that donors regularly used indicators of contraceptive uptake and use to capture family planning success in the period of FP2020, but did not use indicators that captured the fulfilment of individuals' reproductive and contraceptive intentions.

Although our analysis focused specifically on donors, the implications of these findings are relevant for all actors within the family planning community, who hold a shared history and adopt programmatic frameworks built around the same benchmarks. As the community looks to realise an FP2030 agenda enshrined in the principles of voluntarism, informed choice and agency, we encourage all actors to reflect on their genuine motives for funding family planning and how they might better align their rhetoric with their practice. At a time where there is growing recognition of the need for transparency across the international development sector, we encourage the community, and donors in particular, to be clear about the rationales behind their family planning programmes and the means through which they intend to achieve them. For those whose intention is to fund and implement truly women-centred family planning, we ask that they invest in radically rethinking what family planning that is grounded in fulfilling the reproductive and contraceptive intentions of all women might look like, and which desires and choices family planning programmes might recognise as valid. In particular, we call on the whole family planning community to engage in an ongoing and mainstream conversation about redesigning programmatic outcome indicators around the key pillars of FP2030—voluntarism, agency, and choice—to truly put their money where their mouth is.

## Data Availability

The original contributions presented in the study are included in the article, further inquiries can be directed to the corresponding author.

## References

[1] Hartmann B. Reproductive rights and wrongs: The global politics of population control. Boston: South End Press (1995).

[2] Seltzer JR. The origins and evolution of family planning programs in developing countries. Santa Monica, CA: RAND Corporation (2002).

[3] Bradley SEK Casterline JB. Understanding unmet need: history, theory, and measurement. Stud Fam Plann. (2014) 42(2):123–50. 10.1111/j.1728-4465.2014.00381.x PMC4369378 24931072

[4] Berer M. Population and family planning policies: women-centred perspectives. Reprod Health Matters. (1993) 1(1):4–12. 10.1016/0968-8080(93)90057-Z

[5] International Women's Health Coalition. “Women's declaration on population policies”. In: Sen G German A Chen LC, editors. Population policies reconsidered: health, empowerment and rights. Cambridge: Harvard University Press (1994). p. 31–4.

[6] United Nations Population Fund. *Programme of Action, Adopted at the International Conference on Population and Development, Cairo, 5-13 September 1994* (2014). Available at: https://www.unfpa.org/sites/default/files/pub-pdf/programme_of_action_Web%20ENGLISH.pdf (Accessed 28 November 2022).

[7] Family Planning 2020. Family Planning. (2020). Available at: https://www.familyplanning2020.org/ (Accessed 11 September 2019).

[8] RamaRao S Jain AK. Aligning goals, intents, and performance indicators in family planning service delivery. Stud Fam Plann. (2015) 46(1):97–104. 10.1111/j.1728-4465.2015.00017.x 25753061

[9] Hendrixson A. Population control in the troubled present: the ‘120 by 20’ target and implant access program. Dev Change. (2018) 50(3):786–804. 10.1111/dech.12423

[10] Senderowicz L. “I was obligated to accept”: a qualitative exploration of contraceptive coercion. Soc Sci Med. (2019) 239:112531. 10.1016/j.socscimed.2019.112531 31513932

[11] Bendix D Foley EE Hendrixson A Schultz S. Targets and technologies: Sayana Press and Jadelle in contemporary population policies. Gender Place Culture. (2020) 27(3):351–69. 10.1080/0966369X.2018.1555145

[12] Senderowicz L. Contraceptive autonomy: conceptions and measurement of a novel family planning indicator. Stud Fam Plann. (2020) 51(2):161–76. 10.1111/sifp.12114 32358789

[13] World Health Organisation. Unmet need for family planning (%). World Health Organisation. Available at: https://www.who.int/data/gho/indicator-metadata-registry/imr-details/3414 (Accessed 18 December 2022).

[14] Senderowicz L Maloney N. Supply-side versus demand-side unmet need: implications for family planning programs. Popul Dev Rev. (2022) 48(3):689–722. 10.1111/padr.12478 36578790 PMC9793870

[15] Short Fabic M. What do we demand? What do we demand? Responding to the call for precision and definitional agreement in family planning's “demand” and “need” jargon. Glob Health Sci Pract. (2022) 10(1):e2200030. 10.9745/GHSP-D-22-00030 35294394 PMC8885353

[16] Speizer IS Bremner J Farid S. Language and measurement of contraceptive need and making these indicators more meaningful for measuring fertility intentions of women and girls. Glob Health Sci Pract. (2022) 10(1):e2100450. 10.9745/GHSP-D-21-00450 35294385 PMC8885354

[17] Family Planning 2030. The FP Agenda, Family Planning 2030. Available at: https://fp2030.org/fp-agenda (Accessed 15 September 2022)

[18] IntraHealth International. Ouagadougou partnership coordination unit. IntraHealth International. Available at: https://www.intrahealth.org/projects/ouagadougou-partnership-coordination-unit (Accessed 28 November 2022).

[19] Foucault M. The archaeology of knowledge and the discourse on language. London: Tavistock Publications Limited (1972).

[20] Foucault M. Discipline and punish: the birth of the prison. London: Allen Lane (1977).

[21] Burman E Parker I. Discourse analytic research: repertoires and readings of text in action. London: Routledge (2016).

[22] Brisbois B Plamondon K. The possible worlds of global health research: an ethics-focused discourse analysis. Soc Sci Med. (2018) 196:142–9. 10.1016/j.socscimed.2017.11.034 29182962

[23] Braun V Clarke V. “Thematic analysis”. In: Cooper H Camic PM Long DL Panter AT Rindskopf D Sher KJ, editors. APA Handbook of research methods in psychology. Vol 2, research designs: quantitative, qualitative, neuropsychological, and biological. Washington DC: American Psychological Association (2012). p. 57–91.

[24] United Nations Department for Economic and Social Affairs. The 17 goals. United Nations Department for Economic and Social Affairs. Available at: https://sdgs.un.org/goals (Accessed 19 December 2022).

[25] World Health Organisation. Demand for family planning satisfied—modern methods (%). World Health Organisation. Available at: https://www.who.int/data/gho/indicator-metadata-registry/imr-details/3813 (Accessed 19 December 2022).

[26] Cornwall A. Beyond “empowerment lite”: women's empowerment, neoliberal development and global justice. Cadernos Pagu. (2018) 52:e185202. 10.1590/18094449201800520002

[27] Bendix D Schultz S. The political economy of family planning: population dynamics and contraceptive markets. Dev Change. (2017) 49(2):259–85. 10.1111/dech.12363

[28] Bhatia R Sasser JS Ojeda D Hendrixson A Nadimpally S Foley EE. A feminist exploration of ‘populationism’: engaging contemporary forms of population control. Gender Place Culture. (2020) 27(3):333–50. 10.1080/0966369X.2018.1553859

[29] Nandagiri R. What's so troubling about ‘voluntary’ family planning anyway? A feminist perspective. Popul Stud. (2021) 75(sup1):221–34. 10.1080/00324728.2021.1996623 34902284

[30] Eyben R Napier-Moore R. Choosing words with care? Shifting meanings of women's empowerment in international development. Third World Q. (2009) 30(2):285–300. 10.1080/01436590802681066

[31] Lewis D Mosse D. Development brokers and translators: The ethnography of aid agencies. West Hartford, CT: Kumarian Press (2006).

[32] Cornwall A. Buzzwords and fuzzwords: deconstructing development discourse. Dev Pract. (2007) 17(4/5):471–84. 10.1080/09614520701469302

[33] Goddard V. Gender, Agency and Change: Anthropological Perspectives. London: Routledge (2000).

[34] Mahmood S. Feminist theory, embodiment, and the docile agent: some reflections on the Egyptian islamic revival. Cult Anthropol. (2001) 16(2):202–36. 10.1525/can.2001.16.2.202

[35] Abu-Lughod L. Do muslim women really need saving? Anthropological reflections on cultural relativism and its others. Am Anthropol. (2002) 104(3):783–90. 10.1525/aa.2002.104.3.783

[36] Cooper D Harries J Myer L Orner P Bracken H Zweigenthal V. “Life is still going on”: reproductive intentions among HIV-positive women and men in South Africa. Soc Sci Med. (2007) 65(2):274–83. 10.1016/j.socscimed.2007.03.019 17451852

[37] Dyer S. The value of children in African countries: insights from studies on infertility. J Psychosom Obstet Gynecol. (2007) 28(2):69–77. 10.1080/01674820701409959 17538814

[38] Samari G. Women's agency and fertility: recent evidence from Egypt. Popul Res Policy Rev. (2017) 36:561–82. 10.1007/s11113-017-9427-3 28804183 PMC5551041

[39] Johnson-Hanks J. On the modernity of traditional contraception: time and the social context of fertility. Popul Dev Rev. (2004) 28(2):229–49. 10.1111/j.1728-4457.2002.00229.x

[40] Rossier C Senderowicz L Soura A. Do natural methods count? Underreporting of natural contraception in urban Burkina Faso. Stud Fam Plann. (2014) 45(2):171–82. 10.1111/j.1728-4465.2014.00383.x 24931074

[41] Spagnoletti BRM Bennett LR Kermode M Wilpopo SA. ‘The final decision is with the patient’: reproductive modernity and preferences for non-hormonal and non-biomedical contraceptives among postpartum middle class women in Yogyakarta, Indonesia. Asian Popul Stud. (2019) 15(1):105–25. 10.1080/17441730.2019.1578532

[42] Drabo S. Beyond ‘family planning’—local realities on contraception and abortion in Ouagadougou, Burkina Faso. Soc Sci. (2020) 9(11):212. 10.3390/socsci9110212

[43] Moroole MA Materechera SA Otang-Mbeng W Aremu AO. Practices, taboos and techniques of indigenous contraception among batswana traditional healers in Ngaka Modiri Molema district, South Africa. Afr J Phys Act Health Sci. (2020) 26(4):427–37. 10.37597/ajphes.2020.26.4.6

